# ACSL3 regulates lipid droplet biogenesis and ferroptosis sensitivity in clear cell renal cell carcinoma

**DOI:** 10.1186/s40170-022-00290-z

**Published:** 2022-10-03

**Authors:** Timothy D. Klasson, Edward L. LaGory, Hongjuan Zhao, Star K. Huynh, Ioanna Papandreou, Eui Jung Moon, Amato J. Giaccia

**Affiliations:** 1 Department of Radiation Oncology, Stanford School of Medicine, Stanford University, Stanford, CA 94305 USA; 2 Department of Radiation Oncology, The Ohio State Comprehensive Cancer Center, Columbus, OH 43210 USA; 3 Oxford Institute for Radiation Oncology, University of Oxford, Old Road Campus Research Building (ORCRB), Roosevelt Drive, Oxford, OX3 7DQ UK

**Keywords:** Clear cell renal cell carcinoma (ccRCC), Lipid droplets, Ferroptosis, Lipid metabolism, Acyl-CoA synthetase 3 (ACSL3), 5-lipoxygenase (5-LOX)

## Abstract

**Background:**

Clear cell renal cell carcinoma (ccRCC), the predominant subtype of kidney cancer, possesses characteristic alterations to multiple metabolic pathways, including the accumulation of cytosolic lipid droplets. However, the pathways that drive lipid droplet accumulation in ccRCC cells and their importance to cancer biology remain poorly understood.

**Methods:**

We sought to identify the carbon sources necessary for lipid droplet accumulation using Oil red O staining and isotope-tracing lipidomics. The role of the acyl-CoA synthetase (ACSL) family members, an important group of lipid metabolic enzymes, was investigated using siRNA and drug mediated inhibition. CTB and XTT assays were performed to determine the effect of ACSL3 knockdown and lipid starvation on ccRCC cell viability and shRNA was used to study the effect of ACSL3 in an orthotopic mouse model. The relationship between ferroptosis susceptibility of ccRCC and ACSL3 controlled lipid metabolism was examined using CTB and FACS-based assays. The importance of 5-LOX in ferroptosis susceptibility in ccRCC was shown with XTT survival assays, and the expression level and predictive value of 5-LOX in TCGA ccRCC data was assessed.

**Results:**

We found that ccRCC cells obtain the necessary substrates for lipid droplet accumulation by metabolizing exogenous serum derived lipids and not through *de novo* lipogenesis. We show that this metabolism of exogenous fatty acids into lipid droplets requires the enzyme acyl-CoA synthetase 3 (ACSL3) and not other ACSL family proteins. Importantly, genetic or pharmacologic suppression of ACSL3 is cytotoxic to ccRCC cells in vitro and causes a reduction of tumor weight in an orthotopic mouse model. Conversely, ACSL3 inhibition decreases the susceptibility of ccRCC cells to ferroptosis, a non-apoptotic form of cell death involving lipid peroxidation. The sensitivity of ccRCC to ferroptosis is also highly dependent on the composition of exogenous fatty acids and on 5-lipoxygenase (5-LOX), a leukotriene producing enzyme which produces lipid peroxides that have been implicated in other cancers but not in ccRCC.

**Conclusions:**

ACSL3 regulates the accumulation of lipid droplets in ccRCC and is essential for tumor growth. In addition, ACSL3 also modulates ferroptosis sensitivity in a manner dependent on the composition of exogenous fatty acids. Both functions of ACSL3 could be exploited for ccRCC therapy.

**Supplementary Information:**

The online version contains supplementary material available at 10.1186/s40170-022-00290-z.

## Introduction

Clear cell renal cell carcinoma (ccRCC) is the most common renal malignancy, afflicting over 73,000 people in the USA annually [1]. The prognosis for ccRCC is poor, particularly when diagnosed at an advanced stage. While patients with localized disease can be treated effectively with surgery, those patients who present with advanced metastatic ccRCC have a variety of therapeutic options including anti-angiogenic tyrosine kinase inhibitors and T cell checkpoint therapies. However, the response to these agents is often limited and transient, and many patients suffer disease progression after an initial therapeutic response. The primary genetic lesion associated with ccRCC is loss or inactivation of the tumor suppressor gene, *VHL*. The *VHL* gene encodes the Von-Hippel Lindau E3 ubiquitin ligase that is responsible for proteasome mediated degradation of the hypoxia inducible factors HIF-1α and HIF-2α [2, 3]. The consequence of VHL loss of function is constitutively stabilized HIF-1α and HIF-2α, a first step in tumor initiation that increases angiogenesis and drives widespread dysregulation of cellular metabolism.

ccRCCs are highly metabolic tumors which are characterized by a “clear cell” appearance that arises due to an excessive accumulation of lipid droplets and glycogen that gives tumor cells a clear cytoplasm after histological preparation. In fact, ccRCC cells have such a propensity for excessive lipid accumulation that the tumors exhibit a fatty yellow appearance. Lipid droplets are dynamic organelles composed of a neutral lipid core of triglycerides and cholesterol esters enveloped by a phospholipid monolayer in which a wide range of enzymes, transporters, and signaling proteins are embedded. Lipid droplets arise from the endoplasmic reticulum and contribute to diverse cellular processes including energy production, membrane synthesis and cellular signaling [4]. However, the molecular mechanisms that govern lipid droplet accumulation in ccRCC tumors are still poorly defined.

A growing body of evidence supports an important pro-tumorigenic role for altered lipid metabolism across many cancer types. Lipid droplets accumulate in cancer cells downstream of oncogene activation, where they can contribute to cellular bioenergetics by supplying fatty acids for beta oxidation [5]. High expression of CD36, a key fatty acid transporter, has been linked directly to epithelial-mesenchymal transition (EMT) and metastatic potential of cancer cells [6]. Beyond directly supporting cellular bioenergetics, lipid droplets are an important source of eicosanoid production which contribute to oncogenesis by directly promoting cancer cell proliferation, and through cross talk with the surrounding stromal tissue and infiltrating immune cells [7, 8]. Lipid droplets are increased during endoplasmic reticulum (ER) stress and they may play a key protective role for cancer cells during the unfolded protein response [9].

Hypoxia is a major driver of altered lipid metabolism in cancer, through both HIF-α-dependent and HIF-α-independent mechanisms. Hypoxia is known to suppress mitochondrial respiration and beta oxidation by decreasing the expression of PGC-1α and CPT-1α [10, 11]. This decrease in fatty acid catabolism can contribute to lipid droplet accumulation by preventing the breakdown of fatty acids. Hypoxia can also directly increase fatty acid synthesis by upregulating key enzymes such as fatty acid synthase (FAS) [12] and can increase flux through additional metabolic pathways that fuel lipid synthesis, including reductive carboxylation of glutamine [13]. Previous studies have suggested that HIF-1α and HIF-2α contribute to lipid droplet accumulation by increasing expression of lipid droplet coat proteins, perilipin 2 (PLIN2) and HILPDA [14]. These proteins act to stabilize lipid droplets and prevent the breakdown of constituent triglycerides, thereby leading to lipid droplet accumulation. In addition, hypoxia has been previously described to upregulate fatty acid uptake in cancer cells through the HIF-1α-induced expression of fatty acid transporters FABP3 and FABP7 which leads to significant intracellular accumulation of lipids in lipid droplets [10, 13, 15]. These findings illustrate how hypoxic signaling induces multifaceted changes in lipid metabolism.

Proximal tubular cells of the renal cortex, which are thought to be the cell of origin in ccRCC, naturally exhibit very high rates of fatty acid oxidation under normal physiological conditions [16]. Activation of the HIF-α transcriptional network downstream of VHL inactivation likely combines, through the mechanisms discussed above, with this intrinsically high level of lipid metabolism to drive the extreme lipid droplet accumulation observed in this tumor type. Developing a better understanding of the mechanisms that drive lipid droplet accumulation in ccRCC may provide foundational insights into the etiology of this malignancy but perhaps more intriguingly may yield new therapeutic strategies that directly or indirectly target abnormal lipid metabolism.

One such possibility would be to target lipid-laden ccRCC cells by inducing lipid peroxidation to induce cell death, thereby taking advantage of a key metabolic abnormality of ccRCC. Ferroptosis, an iron dependent non-apoptotic form of cell death, is associated with accumulation of lipid peroxidation. Ferroptosis is induced through inactivation of the glutathione peroxidase, GPX4, the enzyme responsible for detoxifying lipid hydroperoxides or by functional inhibition of the cystine-glutamate antiporter [17]. Given the dysregulation of lipid metabolism observed in ccRCC, it is reasonable to hypothesize that ccRCC tumors would be particularly susceptible to cell death by ferroptosis. Indeed, early in vitro cell toxicity screens found that ccRCC derived cell lines were among the most vulnerable cancer cell lines to ferroptosis inducers [18, 19]. Interestingly, inducible deletion of GPX4 results in acute renal failure in mice, suggesting that the importance of this pathway for viability in ccRCC may be a retained trait from normal kidney physiology [20]. The impact of abnormal lipid metabolism on susceptibility to ferroptosis, and whether this pathway could be targeted therapeutically in ccRCC remains unclear.

Here, we provide the first evidence that exogenous fatty acids are the primary source for lipid droplet formation in ccRCC. We demonstrate that acyl-CoA synthetase 3 (ACSL3), an enzyme that catalyzes esterification of fatty acids with Coenzyme A, is the essential driver of lipid droplet accumulation in ccRCC. Inhibition of ACSL3 by genetic or pharmacologic means results in pronounced cytotoxicity to ccRCC cells, suggesting that ACSL3 is an essential viability factor for ccRCC cells. We demonstrate that ACSL3-dependent exogenous lipid metabolism renders ccRCC cells sensitive to ferroptosis-inducing agents in a manner that is highly dependent on exogenous lipid supply and the degree of fatty acid saturation. We show that the sensitivity of ccRCC cells to ferroptosis is mediated through arachidonate 5-lipoxygenase (5-LOX), which oxidizes polyunsaturated fatty acids into highly reactive HpETEs that can promote ferroptotic cell death. In summary, our results demonstrate an important role for ACSL3-mediated lipid metabolism in driving a hallmark metabolic phenotype of ccRCC, the accumulation of lipid droplets, and that ACSL3-dependent lipid accumulation maintains ccRCC cell viability while simultaneously sensitizing cells to ferroptotic cell death, making it a compelling therapeutic target for the treatment of ccRCC.

## Results

### Exogenous lipids drive lipid droplet formation in ccRCC

**Fig. 1:**
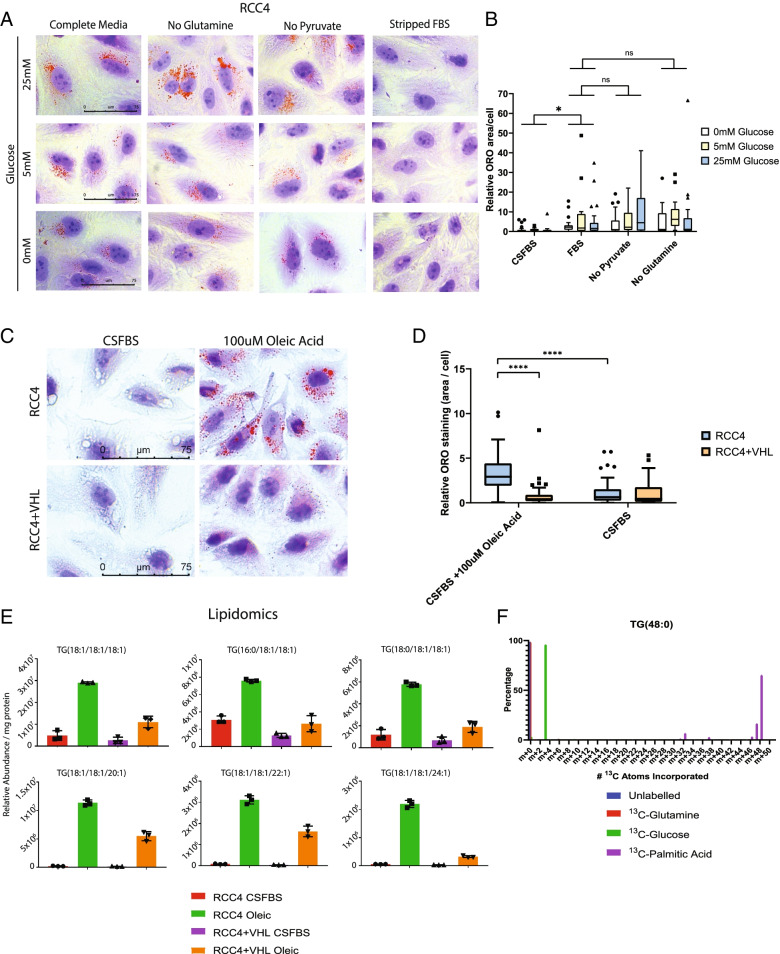
Exogenous lipids are the major source of lipid droplet formation in ccRCC. **A** Oil Red O stained RCC4 cells cultured in media supplemented with or without glucose, glutamine (2 mM), pyruvate (1 mM) and with 10% complete fetal bovine serum (FBS) or charcoal stripped FBS (CSFBS). Images displayed at the same magnification. Imaging was performed with *n* = 2 biological replicates. **B** Quantification of 1A. The box plots summarize the distribution of the amount of Oil Red O staining in each cell, relativized to the average control cell. The whiskers were produced using the Tukey method. Individual points represent values that fall outside the range used in the Tukey method. The edges of the boxes represent the 25th and 75th percentile, while the line in the center of the box represents the mean. Comparisons are multiple comparisons from a two-way ANOVA using Tukey’s multiple comparisons test, comparing the column effect between each group on the *X*-axis. P(CSFBS vs FBS) = 0.0135, p(FBS vs no pyruvate) = 0.3887, p(FBS vs no glutamine) = 0.4771. The row effects (comparisons between each level of glucose) were not significant. *p* > 0.05. **C** Oil Red O stained control and VHL-transduced RCC4 cells cultured in media supplemented with 10% CSFBS ± 100 μM oleic acid for 24 h prior to staining. Images displayed at the same magnification. Imaging was performed with *n* = 2 biological replicates. **D** Quantification of 1C. The box plots summarize the distribution of the percent area of Oil Red O staining in each cell, relativized to the average control cell. The whiskers were produced using the Tukey method. Individual points represent values that fall outside the range used in the Tukey method. The edges of the boxes represent the 25th and 75th percentile, while the line in the center of the box represents the median. Comparisons are multiple comparisons from a two-way ANOVA using Tukey’s multiple comparisons test, comparing all groups. P(RCC4 + oleic vs RCC4 + VHL + oleic) = < 0.0001. P(RCC4 + oleic vs RCC4 + CSFBS) = < 0.0001. **E** LC-MS measurement of triglyceride abundance in control and VHL-transduced RCC4 cells cultured in media supplemented with 10% CSFBS ± 100 μM oleic acid for 24 h prior to staining. *n* = 3, error bars depict standard deviation from the mean. **F** LC-MS ^13^C stable isotope tracing using universally labelled glucose, glutamine, or palmitate to measure fractional labeling of triglycerides containing 3 palmitate acyl groups. The number of labelled carbons measured is depicted on the *x*-axis. *n* = 3, error bars depict standard deviation from the mean

We initiated our studies into the drivers of ccRCC lipid droplet accumulation by determining which metabolites are the predominant drivers of these lipid droplets in ccRCC cells. To do this, we incubated ccRCC cells in media supplemented with different levels of glucose, glutamine, and pyruvate, three metabolites that can contribute to lipid droplet formation by providing precursors for de novo lipogenesis. Notably, cellular lipid droplet content persisted even after removing glutamine or pyruvate, indicating that these carbon sources are dispensable for lipid droplet accumulation in ccRCC cells (Fig. 1A, B). Reducing levels of glucose appeared to lead to a modest reduction in lipid droplet content, but did not lead to a significant quantifiable effect as measured by relative Oil Red O staining (Fig. 1A, B). These results indicate that carbon derived from glycolysis or glutaminolysis are not major contributors to lipid droplet accumulation in ccRCC cells.

In standard cell culture media, exogenous fatty acids are supplied by serum supplementation. To determine whether serum-derived fatty acids contributed to lipid droplet content, we cultured cells in media supplemented with 10% standard fetal bovine serum (FBS), or with 10% charcoal stripped FBS (CSFBS), which is depleted of lipophilic components including free fatty acids, triglycerides, and other lipids. Strikingly, cells cultured in 10% CSFBS exhibited virtually no lipid droplet content, providing strong evidence that access to exogenous lipids is crucial for the lipid droplet accumulation observed in ccRCC cells (Fig. 1A, B).

We hypothesized that if serum lipid content was the primary determinant of lipid droplet abundance, then increasing the fatty acid concentration in the media would result in a corresponding increase in the lipid droplet content of cells. Indeed, VHL-deficient ccRCC lines robustly increased cellular lipid droplet abundance and triglyceride levels in response to supplementation of the media with 100 μM oleic acid (Fig. 1C–E). The ability of these cells to efficiently form lipid droplets in response to fatty acid exposure was dependent on VHL status, since restored VHL expression prevented the increased lipid droplet and triglyceride content, thus providing a direct link between dysregulated lipid metabolism and the major tumor suppressor in ccRCC (Fig. 1C–E). Cholesterol ester (CE) levels were similar between RCC4 with and without VHL and RCC4 cells did not increase in response to oleic acid stimulation, indicating that cholesterol ester regulation in ccRCC cells likely occurs through an alternative mechanism compared to triglycerides (Figure S1A). One hundred micromolar oleic acid also significantly increased lipid droplet content in flow cytometry assays using BODIPY 493/503, a fluorescent neutral lipid dye that can be used to quantify lipid droplets [21] (Figure S1B).

We next used a stable isotope labeling approach to assess the relative contribution of carbons from glucose, glutamine, and fatty acid into triglycerides in ccRCC cells. Supporting our findings that exogenous fatty acids are a key driver of ccRCC lipid droplet formation, we found that carbon from labelled palmitate was rapidly incorporated into triglycerides (Fig. 1F). Palmitate was used for labeling fatty acids because it is the major product of de novo lipogenesis and therefore serves as the most direct point of comparison between fatty acids derived from de novo lipogenesis in the cell and exogenously derived fatty acids. In contrast to the robust palmitate labeling of triglycerides, labelled glutamine produced only a minor incorporation into triglycerides, providing further evidence that glutaminolysis is not a major contributor to ccRCC triglyceride synthesis (Fig. 1F). Interestingly, labelled glucose produced a rapid labelling pattern of m + 3 in triglycerides which is consistent with incorporation of the glycolytic intermediate glycerol-phosphate being incorporated into the backbone of newly synthesized triglycerides (Fig. 1F). In contrast to palmitate labeling, glucose labeling produced little labeling in the mass ranges greater than m + 3, indicating that the contribution of glucose carbon to de novo lipogenesis was not likely to be a major contributor to triglyceride accumulation in ccRCC cells.

These results demonstrate that VHL-deficient ccRCC cells robustly incorporate exogenous lipids into lipid droplets and provide important clues to the origin of the clear cell phenotype in ccRCC. In contrast, de novo lipogenesis did not significantly contribute to lipid droplet biogenesis in the same ccRCC models.

### Acyl-CoA synthetase 3 drives lipid droplet accumulation in ccRCC

**Fig. 2:**
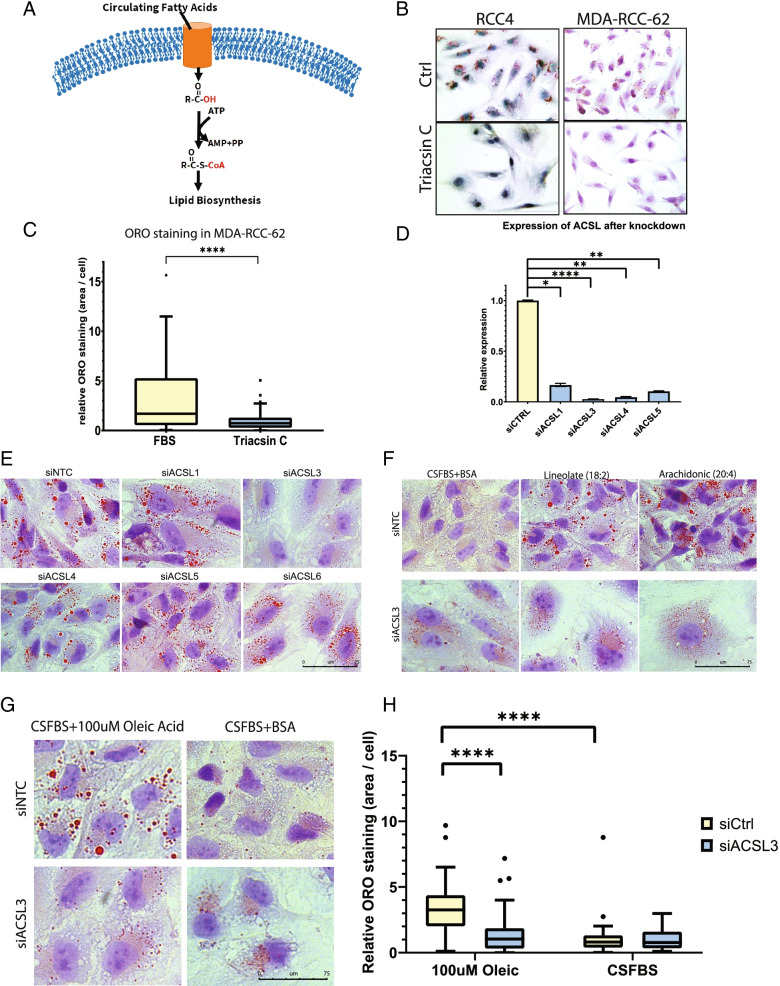
ACSL3 is required for lipid droplet accumulation in ccRCC cells. **A** Acyl-CoA synthetase catalyzes the esterification of free fatty acids with coenzyme A, a step that is necessary for downstream anabolic and catabolic metabolism of fatty acids. **B** Images of Oil Red O stained ccRCC cells treated with the pan-ACSL competitive inhibitor, triacsin C. Images displayed at the same magnification. Imaging was performed with greater than *n* = 3 biological replicates. **C** Quantification of the ORO staining in MDA-RCC-62 cells (see Fig. 1B). The box plots summarize the distribution of the percent area of Oil Red O staining in each cell, relativized to the average control cell. The whiskers were produced using the Tukey method. Individual points represent values that fall outside the range used in the Tukey method. The edges of the boxes represent the 25th and 75th percentile, while the line in the center of the box represents the median. The comparison is an unpaired two-tailed *t* test with 165 degrees of freedom. *P* = < 0.0001. 170 cells were measured over the course of three independent replicate experiments. **D** qPCR measurement of mRNA expression of ACSL subspecies in MDA-RCC-62 cells transfected with either siRNA against the indicated ACSL subspecies or a control scrambled siRNA (siCTRL). ACSL6 was not found to be expressed in ccRCC cells and therefore was not included in this figure. Bar graph represents mean and error bars represent standard deviation. All knockdowns were performed 3 times per experiment in 2 independent experiments. **E** Images of Oil Red O stained ccRCC cells transfected with siRNA against ACSL1, 3, 4, 5, and 6 cultured with 100 μM oleic acid for 24 h. Images displayed at the same magnification. Imaging was performed with *n* = 2 biological replicates. **F** Images of Oil Red O stained ccRCC cells transfected with siACSL3 and cultured with 100 μM linoleic acid, arachidonic acid, or control media for 24 h prior to fixation and staining. Images displayed at the same magnification. Imaging was performed with *n* = 2 biological replicates. **G** Oil Red O stained ccRCC cells treated with either scrambled control siRNA or siRNA against ACSL3, treated with either charcoal stripped FBS or charcoal stripped FBS supplemented with 100 μM oleic acid. Images displayed at the same magnification. Imaging was performed with *n* = 2 biological replicates. **H** Quantification of 2G. The box plots summarize the distribution of the percent area of Oil Red O staining in each cell, relativized to the average control cell. The whiskers were produced using the Tukey method. Individual points represent values that fall outside the range used in the Tukey method. The edges of the boxes represent the 25th and 75th percentile, while the line in the center of the box represents the median. 287 cells were counted over 3 independent replicate experiments. Comparisons are multiple comparisons from a two-way ANOVA using Šídák’s multiple comparisons test to measure the differences between all groups. P(siCtrl + oleic vs siACSL3 + oleic) = < 0.0001. P(siCtrl + oleic vs siCtrl + CSFBS) = < 0.0001

Having identified serum lipids as a major source of lipid droplet synthesis in ccRCC, we next sought to understand the enzymatic mechanism driving the incorporation of exogenous lipids into lipid droplets. Members of the long chain acyl-CoA synthetase (ACSL) enzyme family catalyze the ATP-dependent esterification of free fatty acids with coenzyme A to form fatty acyl-CoA (Fig. 2A). The ACSL-dependent esterification with coenzyme A effectively traps the fatty acid inside of the cell and allows channeling into downstream lipid metabolic pathways, including the synthesis of higher order lipids. Esterification of fatty acids to CoA by ACSL enzymes is also required for their catabolism by beta oxidation. This dual role indicates that the esterification of free fatty acids by ACSL is an essential first step in the metabolism of free fatty acids. To broadly assess the role of ACSL activity in ccRCC lipid droplet abundance, we used triacsin C, a fungal metabolite that acts as a competitive inhibitor of multiple ACSL family members [22]. Consistent with an essential role for ACSL enzymes in lipid metabolism, triacsin C treatment profoundly decreased lipid droplet content in ccRCC cell lines, indicating a critical role for this enzyme family in ccRCC lipid droplet formation (Fig. 2B, C).

The ACSL enzyme family is comprised of 5 members (ACSL1, 3, 4, 5, and 6) with varying substrate specificity and tissue distribution [23]. We used siRNA knockdown to determine whether specific ACSL family members were important for lipid droplet formation in VHL-deficient ccRCC cells when the cells were exposed to exogenous fatty acids. ACSL6 was not found to be expressed at a detectible level in ccRCC cells. We found that genetic suppression of ACSL3, but not the other family members, significantly diminished oleic acid-induced lipid droplet formation in ccRCC cells as indicated by Oil Red O and BODIPY staining (Fig. 2D, E). The requirement for ACSL3 was not specific to oleic acid-induced lipid droplet formation but was required for lipid droplet formation in response to a diverse array of fatty acids of varying acyl chain length and saturation levels (Fig. 2F). Importantly, oleic acid-induced lipid droplet formation in CSFBS cultured ccRCC cells was dependent on ACSL3 expression as measured by quantitation of Oil Red O staining (Fig. 2G, H).

We utilized two approaches to verify that the effects observed for siRNA mediated knockdown of ACSL3 were on target. First, we synthesized and cloned an siRNA-resistant form of ACSL3 in which the siRNA binding sites were mutated in a way that would not alter the amino acid sequence of the encoded protein. Expression of this siRNA resistant mutant form of ACSL3 prevented the ability of siACSL3 transfection to deplete oleic acid-induced lipid droplet formation, verifying that our observations of ACSL3 knockdown on lipid droplet formation were on target (Figure S2A, B). We also individually tested each siRNA oligonucleotide from the pool and found that multiple oligonucleotides were able to suppress lipid droplet synthesis (Figure S2C and S2E). Together, these results confirm our finding that ACSL3 is a critical regulator of lipid droplet synthesis in ccRCC cells.

Surprisingly, while we have provided strong evidence that ACSL3 is an essential factor in lipid droplet synthesis in ccRCC, the mRNA expression levels of ACSL3 were not altered in ccRCC tumors compared to adjacent normal kidney, indicating that it is not a VHL dependent gene (Figure S1C). These results strongly suggest that basal levels of ACSL3 expression are sufficient to drive the lipid droplet phenotype, and that further increased expression in ccRCC is not needed to drive the clear cell phenotype.

### ACSL3 expression and activity is an essential ccRCC viability factor

**Fig. 3:**
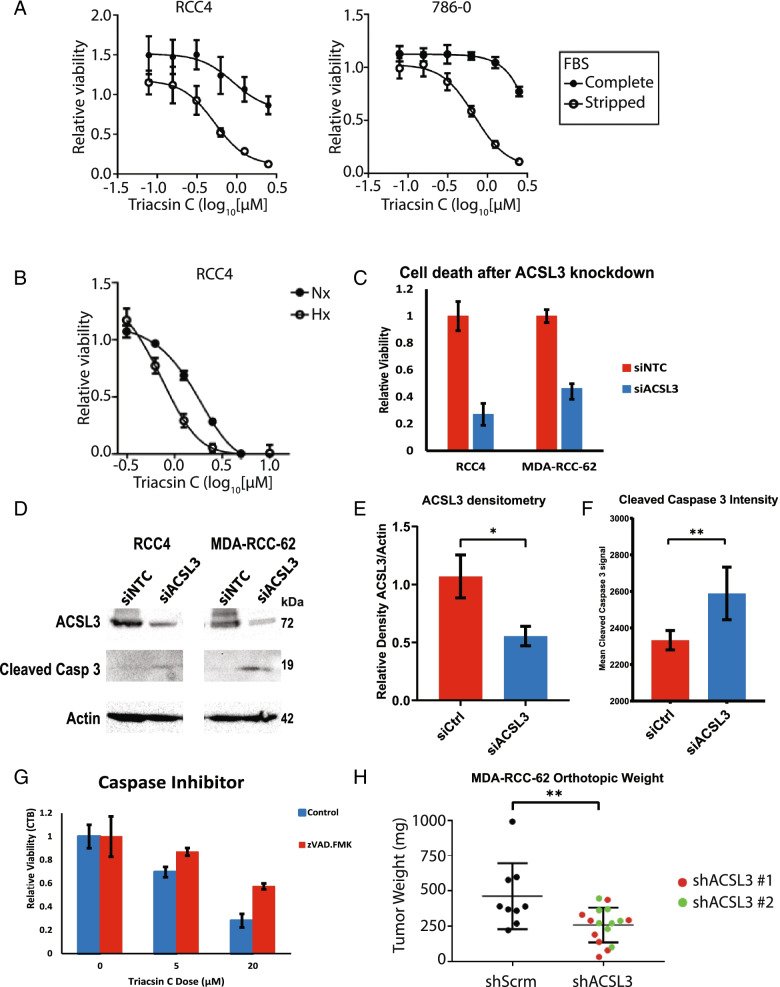
ACSL3 inhibition induces cell death in ccRCC. **A** XTT cell viability assay of ccRCC cells cultured in media supplemented with standard FBS or CSFBS and treated with increasing concentrations of triacsin C. *n* = 3, error bars depict standard deviation from the mean. **B** XTT cell viability assay of ccRCC cells cultured in atmospheric oxygen (21% O_2_, Nx) or hypoxia (0.5% O_2_) and treated with increasing concentrations of triacsin C. *n* = 3, error bars depict standard deviation from the mean. **C** Cell titer blue assay of cell viability in ccRCC cells transfected with siACSL3 or non-targeting control siRNA. *n* = 3, error bars depict standard deviation from the mean. **D** Representative western blots of ACSL3, cleaved caspase 3 and β-actin from ccRCC cells transfected with siACSL3 or non-targeting control siRNA. **E** Densitometry quantifications of ACSL3 western blots using actin as a loading control. *n* = 3, error bars depict standard deviation from the mean. Comparisons are from an unpaired two-tailed Student’s *t* test comparing control cells with cells transfected with siRNA against ACSL3. *p < 0.05. **F** FACS assay of cleaved caspase 3 in ccRCC cells transfected with siRNA against either ACSL3 or non-targeting control. *n* = 3 with 2 technical replicates representing 10,000 cells per technical replicate. Comparisons are from an unpaired two-tailed Student’s *t* test comparing the mean fluorescence intensity of cleaved caspase 3 positive cells in the two groups. **p < 0.01. **G** Cell titer blue viability assay of ccRCC cells pre-treated with the caspase inhibitor, zVAD.FMK followed by treatment with increasing concentrations of triacsin C. **H** Orthotopic ccRCC xenograft model of tumor weight in mice injected with ccRCC cells. Mice were injected with ccRCC cells treated with shRNA against either ccRCC or negative control. *n* = 25. The edges of the boxes represent the 25th and 75th percentile, while the line in the center of the box represents the median. Comparisons are from an unpaired two-tailed Student’s *t* test comparing the control cells with the shACSL3 cells grouped together. **p < 0.01

To determine whether inhibiting ACSL-mediated lipid metabolism was able induce ccRCC cell death, we treated ccRCC cells with triacsin C in standard media, or in media supplemented with CSFBS. Interestingly, while triacsin C treatment alone reduced the viability of ccRCC cells, culture in CSFBS supplemented media significantly enhanced the cytotoxicity of the ACSL inhibitor (Fig. 3A). Because triacsin C is a competitive inhibitor of ACSLs, the reduction of exogenous lipid substrate through culturing cells with CSFBS leads to full inhibition at lower levels of triacsin C. Additionally, the increased sensitivity of ccRCC cells to triacsin C when cultured in lipid depleted media could be due to inhibition of multiple ACSL enzymes, which are active in ccRCC even if they are not required for lipid droplet accumulation, or due to inhibiting lipid droplet independent functions of ACSL3 and the ACSL family. Interestingly, exposure to hypoxia, which is known to induce dependency on exogenous fatty acids via steroyl-Coa desaturase inhibition [24], also heightened ccRCC cell sensitivity to triacsin C (Fig. 3B). Since our previous results showed ACSL3 to be especially important for ccRCC cells, we sought to determine whether blocking ACSL3 individually using siRNA could also affect viability. Consistent with our results showing ACSL3 to be the most essential ACSL family member for ccRCC lipid metabolism, targeting ACSL3 with siRNA lead to a reduction in cell viability compared with controls (Fig. 3C). This reduced viability was likely due to induction of apoptosis as we observed a concomitant accumulation of cleaved caspase 3 in ACSL3 knockdown ccRCC cells (Fig. 3D–F). To determine whether triacsin C-induced cytotoxicity was dependent on apoptosis, we pre-treated cells with the caspase inhibitor zVAD.fmk and found that this partially protected ccRCC cells from triacsin C-induced cell death (Fig. 3G). Together, these results indicate that ccRCC cells depend upon ACSL3 expression and activity to maintain viability, and that this is likely due to a dependence on exogenous fatty acid metabolism for viability.

Having established that ACSL3 function is important for ccRCC cell line viability in vitro, we next sought to determine if ACSL3 was also important for ccRCC tumor biology in an in vivo model. To this end, ccRCC cells were transfected with one of two short-hairpin RNA (shRNA) targeting ACSL3 or a scrambled negative control. Both shRNA targeting ACSL3 produced robust knockdown of ACSL3 mRNA expression compared with the control shRNA (Figure S2D). Rag2−/−IL2rg−/− double knockout mice were then injected with a collagen plug containing the transfected ccRCC cells under the renal capsule. These tumors were then weighed 6 weeks later. Consistent with our previous in vitro results, ccRCC cells with ACSL3 silenced by transduction with shRNA displayed reduced growth in this orthotopic murine tumor model (Fig. 3H). These results demonstrate that, in addition to being essential for ccRCC cell viability in vitro, ACSL3 function is similarly important for tumor growth and tumor cell viability in vivo.

### Metabolism of exogenous lipids sensitizes VHL-deficient ccRCC cells to ferroptosis

Our data indicate that ACSL3 is critical for lipid droplet formation and that loss of ACSL3 and lipid droplets decreases ccRCC viability through apoptotic cell death, raising the possibility of direct therapeutic targeting of ACSL3 in ccRCC. Another possibility is to target lipid metabolism indirectly by exploiting vulnerabilities, such as enhanced lipid peroxidation, that accompany this dysregulated metabolic state. Accumulation of lipid peroxidation can induce ferroptosis, a non-apoptotic form of programmed cell death that can be induced by treatment with inhibitors of glutathione metabolism or the glutathione peroxidase, GPX4 [17]. Notably, polyunsaturated fatty acids (PUFA) are highly prone to lipid peroxidation and the generation of PUFA-containing phospholipids is necessary for ferroptosis [25, 26]. Human cells are unable to synthesize PUFAs de novo and these fatty acids must instead be taken up from circulation by cells. These factors point to an important role for the metabolism of exogenous fatty acids, and PUFAs in particular, in determining cellular liability to ferroptosis and suggest that cells with a high rate of fatty acid uptake from the serum may be particularly sensitive to ferroptosis. Supporting the hypothesis that uptake of PUFAs promotes susceptibility to ferroptosis, it was recently demonstrated that colon cancer cells exposed to PUFAs were sensitive to ferroptosis inducing agents, while the same cells exposed to the monounsaturated oleic acid were protected from ferroptosis [27].

**Fig. 4:**
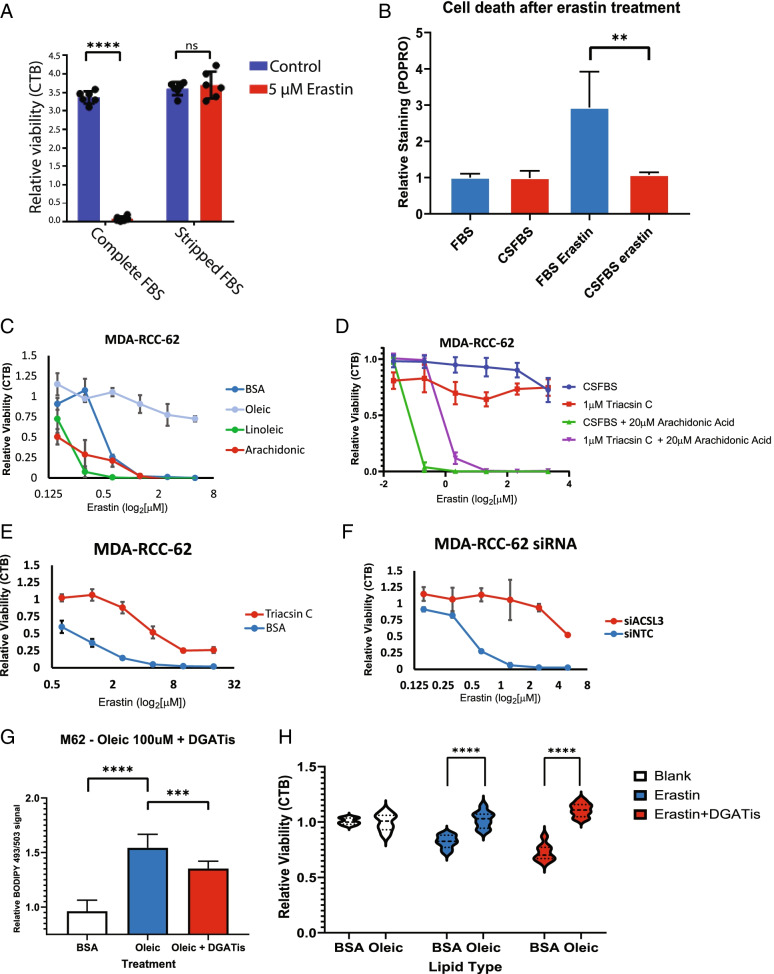
Metabolism of exogenous lipids sensitizes ccRCC cells to ferroptosis inducers. **A** Cell titer blue viability assay of MDA-RCC-62 cells cultured in media supplemented with standard FBS or CSFBS and treated with 5 μM erastin. *n* = 3 biological replicates, error bars depict the standard deviation from the mean. Comparisons are multiple comparisons from a one-way ANOVA using Šídák’s multiple comparisons test to measure the difference between erastin and untreated cells within each media group. ****p < 0.0001. **B** FACS assay of MDA-RCC-62 cells stained with POPRO membrane permeable dye to mark dying cells. Cells were cultured in media supplemented with either standard FBS or CSFBS and treated with 2.5 μM erastin. Bars represent the mean value and error bars represent the standard deviation. Results are from twelve replicates performed over two independent experiments. Comparisons are multiple comparisons from a one-way ANOVA using Šídák’s multiple comparisons test to measure the differences between all groups. *F* = 14.54 with 8 degrees of freedom. P(FBS + erastin vs CSFBS + erastin) = 0.0046. **C** Cell titer blue viability assay of MDA-RCC-62 cells cultured with BSA, or BSA + 100 μM oleic acid, linoleic acid, or arachidonic acid and treated with the indicated concentration of erastin. **D** Cell titer blue viability assay of MDA-RCC-62 cells cultured in media supplemented with 10% CSFBS + BSA, or BSA + 20 μM arachidonic acid, or BSA + 20 μM arachidonic acid + 1 μM triacsin. *n* = 4 biological replicates, error bars depict the standard deviation from the mean. **E** Cell titer blue assay of MDA-RCC-62 cells treated with 1 μM triacsin C or vehicle control and increasing concentrations of erastin. *n* = 3 biological replicates, error bars depict the standard deviation from the mean. **F** Cell titer blue viability assay of MDA-RCC-62 cells transfected with siACSL3 or non-targeting control siRNA and treated with increasing concentrations of erastin. *n* = 3 biological replicates, error bars depict the standard deviation from the mean. **G** FACS analysis of BODIPY 493/503 fluorescent intensity in ccRCC cells cultured with only BSA, added 100 μM oleic acid, or oleic acid and DGAT inhibitor cocktail (DGATis). Bars represent the mean value and error bars represent the standard deviation. Results are from 30 replicates performed over 4 independent experiments. Comparisons are multiple comparisons from a one-way ANOVA using Šídák’s multiple comparisons test to measure the differences between all groups. *F* = 86.21 with 27 degrees of freedom. P(BSA vs oleic) = < 0.0001. P(oleic vs oleic + DGATis) = 0.0005. **H** Cell titer blue viability assay of MDA-RCC-62 cells cultured with either BSA or 100 μM oleic acid alone or supplemented with either erastin or erastin and DGATis. Results are presented as a violin plot, where the width of the shape shows the distribution of the data. The dashed line represents the median where the dotted lines represent the quartiles. Results are from 48 replicates performed over 2 independent experiments. Comparisons are multiple comparisons from a two-way ANOVA using Šídák’s multiple comparisons test to measure the differences between oleic and BSA within each group (row effect). P(BSA + erastin vs oleic + erastin) = < 0.0001. P(BSA + erastin + DGATis vs oleic + erastin + DGATis) = < 0.0001

Interestingly, ccRCC cells have been reported to be highly susceptible to ferroptosis-inducing agents such as erastin, an inhibitor of the cystine-glutamate antiporter system Xc, which blocks the synthesis of glutathione, an antioxidant used to detoxify lipid peroxides [18]. Direct cysteine deprivation has also been shown to induce necrosis in ccRCC cells in a VHL dependent manner [28]. To determine whether ccRCC sensitivity to ferroptosis induction was dependent upon exogenous lipid metabolism, we cultured ccRCC cells in standard media conditions, or in lipid-deficient media supplemented with charcoal stripped FBS. We found that ccRCC cells cultured in standard media were highly susceptible to the ferroptosis inducing agent, erastin, whereas the same cells cultured in lipid deficient media were almost completely resistant (Fig. 4A). ccRCC cells cultured in CSFBS supplemented media and treated with erastin showed less cell death than cells cultured in standard media and treated with erastin as measured by a fluorescence-based flow cytometry assay (Fig. 4B).

The fatty acid saturation level of lipids is thought to be an important determinant of ferroptosis sensitivity, with monounsaturated fatty acids promoting ferroptosis resistance and polyunsaturated fatty acids promoting sensitivity [25, 27]. To determine whether the saturation level of the exogenous fatty acids was important for ferroptosis sensitivity in ccRCC, we cultured ccRCC cells in the presence of either saturated or unsaturated fatty acids, or BSA alone and exposed them to increasing concentrations of erastin. Supporting our hypothesis that exogenous PUFAs increase sensitivity of ccRCC cells to ferroptosis, we found that cells cultured in linoleic or arachidonic acid were greatly sensitized to ferroptotic cell death (Fig. 4C). In contrast, cells cultured in the monounsaturated oleic acid were highly resistant to ferroptosis induction (Fig. 4C). The sensitizing effect of arachidonic acid on erastin-induced lethality could also be partially abrogated by triacsin C treatment, reinforcing the important role of ACSL enzymatic activity in exogenous fatty acid metabolism (Fig. 4D). These results suggest that the degree of unsaturated fatty acid metabolism dictates the susceptibility of ccRCC cells to erastin-induced ferroptosis.

ACSL4 has been previously reported as an essential factor for ferroptosis induction [16]. Given our observations that the incorporation of exogenous fatty acids into lipid droplets was dependent upon ACSL3, we tested whether ACSL expression or activity was important for erastin-induced ferroptosis in ccRCC. Pretreatment of ccRCC cells with the ACSL inhibitor, triacsin C, led to a significant reduction in sensitivity to ferroptosis (Fig. 4D, E). siRNA-mediated suppression of ACSL3 expression alone rendered ccRCC cells resistant to ferroptosis induction, supporting an important role for ACSL3 in this process (Fig. 4F). Together, these results indicate that ACSL3-dependent metabolism of exogenous polyunsaturated fatty acids renders ccRCC cells sensitive to ferroptosis inducing agents. Importantly, the critical role of ACSL3 in erastin-induced ferroptosis does not preclude a role for ACSL4 or other ACSL family members in ferroptosis.

It has been previously shown that ACSL3 activity was responsible for the protective effect of oleic acid against ferroptosis induction in colon cancer cells independently of lipid droplet synthesis [27]. Because ACSL3 mediated lipid metabolism is responsible for the excessive lipid droplet production phenotype seen in ccRCC, we asked whether lipid droplets were required for lipid protection against ferroptosis in ccRCC cells. To do this, we blocked lipid droplet production by inhibiting diglyceride acyltransferase (DGAT), an enzyme downstream of ACSL3 that catalyzes the final reaction in triglyceride synthesis (Fig. 4G). We found that while DGAT inhibition led to a modest reduction in oleic acid-induced lipid droplet formation, the protective effect of oleic acid against ferroptosis was maintained (Fig. 4H). These results are consistent with a previous report that DGAT inhibitors do not prevent ferroptosis and indicate that the role of ACSL3 in ferroptosis may be in part independent of the formation of lipid droplets.

### 5-Lipoxygenase pathway drives sensitivity to ferroptosis

**Fig. 5:**
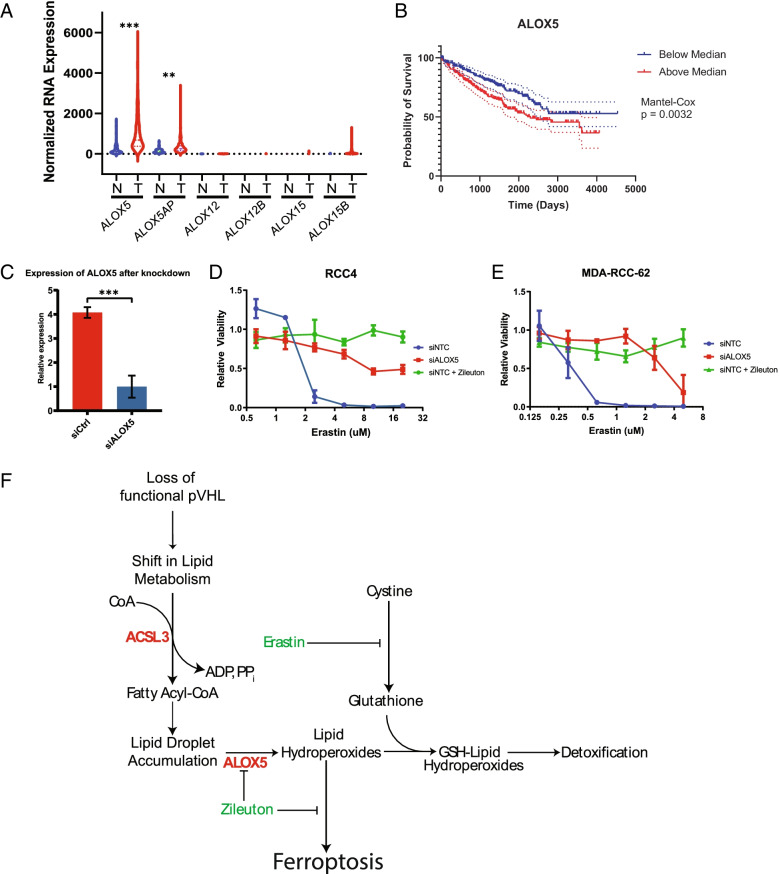
5-lipoxygenase drives ccRCC migration phenotype and promotes sensitivity to ferroptosis. **A** ALOX family gene expression in ccRCC tumor biopsies (denoted as T) compared to non-malignant renal cortex (denoted as N). Data curated from TCGA KIRC dataset. Comparisons are multiple comparisons from a one-way ANOVA using Šídák’s multiple comparisons test to measure the differences between all groups. 11 degrees of freedom. P(ALOX5 N vs ALOX5 T) = < 0.0001. P(ALOX5AP N vs ALOX5AP T) = 0.002. **B** Kaplan-Meyer plots of overall survival in patients expressing high (upper quartile) or low expression of 5-LOX pathway genes. Dashed lines represent 95% confidence intervals. **C** qPCR measurement of relative mRNA expression of ALOX5 in ccRCC cells transfected with siRNA against either ALOX5 or a control scrambled siRNA (siCTRL). Bar graph represents mean and error bars represent standard deviation. *n* = 2 with 3 technical replicates per experiment. Comparisons are from an unpaired two-tailed Student’s *t* test. ***p < 0.001. **D**, **E** Cell titer blue viability assay of ccRCC cells (M62 or RCC4 cells as indicated) cultured with 5-LOX inhibitor zileuton or transfected with siALOX5 and increasing concentrations of erastin. *n* = 3 biological replicates, error bars depict the standard deviation from the mean. **F** Summary model of the role of ACSL3 and 5-LOX in ccRCC as well as compounds effecting the system. Loss of functional pVHL leads to a change in metabolism that leads to an increase in lipid uptake as well as a decrease in beta oxidation of lipids, resulting in lipid droplet accumulation in the cytoplasm. ACSL3 is required to process lipids which eventually leads to downstream PUFA metabolism that leads to the production of lipid hydroperoxides. These lipid peroxides can sensitize the cell to ferroptosis if the cell is unable to properly detoxify them

It has been previously reported that lipoxygenase enzymes such as 5-LOX and 15-LOX generate the lipid hydroperoxides that drive ferroptotic cell death [25, 29, 30]. These enzymes metabolize arachidonic acid and other polyunsaturated fatty acids to produce a diverse array of leukotrienes that signal in autocrine and paracrine mechanisms to induce pleiotropic effects on tumor biology, including promoting proliferation and migration, as well as signaling to surrounding immune and stromal cells [31]. To determine whether this pathway might be active in ccRCC, we compared gene expression profiles of LOX family members in ccRCC compared to normal renal cortex samples from TCGA datasets. Strikingly, we found that several LOX family genes were upregulated in ccRCC compared to the expression levels in normal renal cortex (Fig. 5A). Furthermore, increased expression of 5-LOX (gene symbol *ALOX5*), a key enzyme in the synthesis of leukotrienes and other eicosanoids in the tumors of ccRCC patients was associated with decreased overall survival (Fig. 5B). 5-LOX has previously been shown to be important for pathophysiological processes including growth, survival and migration in several cancers including colon [32] and prostate [33].

To determine whether 5-LOX pathway activity was required for ferroptosis sensitivity in ccRCC cells, we tested whether the FDA approved 5-LOX inhibitor, zileuton, could block erastin-induced ferroptosis. Confirming an important role for 5-LOX activity in ferroptosis, we found that zileuton significantly reduced lethality in ccRCC cells treated with erastin (Fig. 5D, E). In addition, knockdown of 5-LOX expression by siRNA (Fig. 5C) also protected ccRCC cells from erastin-induced ferroptosis (Fig. 5D, E). This data indicates that the upregulation of this pathway in ccRCC is promoting the tumor phenotype while simultaneously rendering tumors vulnerable to ferroptosis.

## Discussion

 The results presented in this study bring new insight into the origins of the clear cell phenotype in ccRCC, a metabolic feature that provides the namesake for this tumor type. Our results demonstrate that ccRCC cells rely on access to exogenous fatty acids to drive lipid droplet deposition through an ACSL3 dependent mechanism. We provide evidence that exogenous PUFA lipid metabolism by ccRCC cells, driven by ACSL3, renders these cells exquisitely sensitive to ferroptosis-inducing agents such as erastin. Our finding that the lipid laden clear cell phenotype is tightly linked to ferroptosis susceptibility is intriguing because it suggests that this hallmark metabolic feature of ccRCC tumors could represent a promising therapeutic vulnerability.

Importantly, we provide evidence that inhibition of ACSL3 induces cell death in ccRCC cells, suggesting that this pathway is essential for the survival of ccRCC cells. ACSL3 inhibition accordingly also reduced tumor growth in an orthotopic mouse model, indicating that this pathway is also vital for cell viability in vivo. Future efforts into how ACSL3-mediated lipid metabolism supports ccRCC viability will be essential to better understand how ccRCC cells harness enhanced lipid metabolism for survival. Given the previously reported suppression of mitochondrial respiration and beta oxidation in ccRCC, it is unlikely that increased levels of lipids are necessary for survival because they support required cellular bioenergetics [10, 11]. It has also been reported that ccRCC lipid droplets rely on the lipid coat protein PLIN2 and that suppression of lipid droplets by inhibiting PLIN2 results in susceptibility to inducers of ER stress [14], implying a similar result may occur after reducing lipid droplets through ACSL3 inhibition. While the mechanisms by which fatty acid metabolism contribute to ccRCC tumor biology remain elusive, the clear role for ACSL3 in driving lipid droplet deposition and maintaining ccRCC viability highlights the potential of targeting this pathway. While triacsin C is a competitive inhibitor of multiple ACSL family members, development of a specific ACSL3 inhibitor could enhance the therapeutic efficacy of such a strategy, while mitigating some of the expected side effects of blocking all ACSL family members.

Our data suggest that ccRCC cells exist in a metabolic state in which exogenous lipids supply lipid droplet biogenesis while also supplying polyunsaturated fatty acids to feed into the 5-LOX pathway. Lipid hydroperoxides that are generated by the 5-LOX pathway represent a potential liability to the ccRCC cell that must be balanced by the detoxification pathway mediated by GPX4. Accumulation of lipid hydroperoxides therefore potentially renders ccRCC cells exquisitely sensitive to direct and indirect inhibitors of the GPX4 pathway, particularly in the presence of 5-LOX substrates like arachidonic acid. These results are in line with other reports that fatty acid saturation levels alter the threshold to ferroptosis inducing agents [27, 34]. A major research question going forward will be whether it is possible to remodel the tumor lipidome through a tailored diet rich in polyunsaturated fatty acids or through pharmaceutical intervention. Based on our results, achieving an increased level of polyunsaturated fatty acids would sensitize ccRCC cells to ferroptosis.

In summary, we propose the following chain of events that occur in ccRCC. Loss of the VHL tumor suppressor gene results in elevated levels of HIF-1 and HIF-2 expression and activity. Two major metabolic consequences of HIF activation are inhibition of mitochondrial metabolism and an increase in free fatty acid transport into cells. Both events are expected to increase the pool of free fatty acids, which ACSL3 metabolizes into Fatty Acyl CoAs, thereby activating the fatty acid for downstream metabolism. PUFA lipids processed in this way can then be packaged into lipid droplets or be metabolized by 5-LOX into lipid hydroperoxides. ccRCC cells are likely able to maintain homeostasis under these conditions, but the increased intracellular concentration of lipid hydroperoxides heightens the cells requirement for the GPX4 lipid peroxide detoxification pathway, thus rendering the cells highly susceptible to direct or indirect inhibitors of GPX4 activity. Loss of ACSL3, inhibition of 5-LOX, or lipid starvation are therefore protective against ferroptosis inducers (Fig. 5F).

Our results indicate that the ACSL3 driven alterations in ccRCC lipid metabolism could be therapeutically targeted in several ways. ACSL3 appears to function as a central regulator of ccRCC lipid metabolism. Importantly, ACSL3 inhibition is cytotoxic to ccRCC cells, indicating that targeting ACSL3 directly will be a promising therapeutic strategy. Taken together, altered lipid metabolism is tightly intertwined with ccRCC tumor biology, and that developing agents that can target these pathways hold great promise for the treatment of this deadly disease.

## Methods

### Cell lines and reagents

The cell lines used in this study (RCC4, MDA-RCC-62) have been previously described [35, 36]. All cells were routinely verified to be free of mycoplasma contamination using the MycoAlert bioluminescence mycoplasma detection kit according to manufacturer’s recommended protocol (Lonza, LT07-118). Cells were cultured in standard DMEM media (Gibco) supplemented with 10% fetal bovine serum unless otherwise indicated. Charcoal stripped FBS and normal pooled human serum were purchased from Gemini Bio (100–110). All fatty acids were purchased from Sigma-Aldrich and were complexed to fatty acid BSA by incubating in 10% BSA w/v dissolved in PBS for 1–4 h at 37 °C prior to supplementation into culture medium. For palmitate-BSA conjugation, a palmitate solution was first prepared by dissolving palmitate in ethanol and adding this ethanolic solution to the BSA for conjugation. Triacsin C (Sigma-Aldrich, T4540), erastin (Sellekchem, S7242), zileuton (Sellekchem, S1443), and ferrostatin-1 (Sigma-Aldrich, SML0583). siRNA SmartPools (Dharmacon) were transfected into cells using RNAiMAX (Invitrogen) according to manufacturer’s protocol. DGAT inhibition was achieved by a combination of two DGAT inhibitors purchased from Sigma-Aldrich: T863 (Cat# SML0539) and PF-06424439 (Cat# PZ0233) were added into cell culture media at 20 μM and 10 μM respectively as described previously [27]. Two different shRNA against ACSL3 were designed using the following target sequences: (1) GCTGTGTAACAGTTGTGAAAT, (2) CCTGGATGTGATACTTTAGAT. For western blotting, the following antibodies were used: anti-ACSL3 (Santa Cruz Biotechnology Cat# sc-166374), anti-cleaved caspase 3 (cell signaling cat#9661), and anti-actin (Sigma-Aldrich A5441).

### Oil Red O staining

Oil Red O (Sigma-Aldrich, O0625) was dissolved in propylene glycol by heating to 95 °C and filtering through Whatman filter paper to a final concentration of 0.5% w/v. Immediately prior to staining, the Oil Red O solution was filtered through a 0.45 μm syringe filter to remove any residual particulate from the solution. Prior to staining, cells cultured on coverslips were fixed in 4% paraformaldehyde, rinsed in PBS, and equilibrated in 100% propylene glycol for 2 min at room temperature. Cells were stained in Oil Red O for 1 h at room temperature and rinsed in 85% propylene glycol to differentiate the staining. After rinsing in PBS, cells were counterstained with Hematoxylin, Gill’s no. 1 (Sigma-Aldrich, GHS132) for 1 min, rinsed, and mounted onto glass slides using Fluoromount-G (SouthernBiotech, 0100-01). Imaging was performed on a Leica DMi8 inverted microscope. Image analysis was done in ImageJ by converting images to the YIQ color space before thresholding and measuring the area of lipid droplets per area of cell.

### In vitro lipidomics sample preparation

For isotope tracing experiments, stable isotope labelled metabolites were ordered from Sigma and supplemented in to a basal DMEM formulation containing no glucose, glutamine, or serum supplementation to the indicated final concentrations (25 mM glucose, 2 mM glutamine, 50 μM palmitate). Cells were cultured with media supplemented with unlabelled metabolites or individual stable isotope labelled metabolites for 18 h prior to lipid extraction. Samples were prepared for lipidomics according to previously described methods [37]. Briefly, cells were scraped into 1.5 mL of ice-cold methanol. The methanol/cell mixture was transferred to glass culture tubes and 5 mL of MTBE was added. This mixture was shaken for 1 h at room temperature. After incubation, 1.25 mL of water was added to induce phase separation followed by an additional 10 min of shaking. This mixture was centrifuged at 1000×*g* for 10 min and the upper phase containing hydrophobic lipid species was immediately transferred to new glass culture tube. This solution was evaporated to dryness and resuspended in 9:1 methanol:toluene prior to LC-MS analysis.

### LCMS method

Lipidomics samples were analyzed using a 6545 QTOF mass spectrometer (Agilent) operated in positive mode, coupled with an Infinity 1290 UPLC (Agilent). The source settings for the mass spectrometer were as follows: drying gas temperature 300 °C; drying gas flow: 11 L/min; sheath gas temperature 300 °C; sheath gas flow 11 L/min; Vcap 3500 V; Octopole RF 750; fragmentor 150V; nozzle voltage 0 V; nebulizer 35 psig; skimmer 65 V. Data was acquired from 65 to 1000 m/z at an acquisition rate of 1 spectra/s. The LC method used a ZORBAX Eclipse Plus RRHD C18, 2.1 × 100 mm, 1.8 μm column (Agilent, 959793-902) maintained at 50 °C and a flow rate of 0.35 mL/min. Mobile phase A (50:10:40 isopropanol:methanol:water + 5 mM ammonium acetate + 0.1% acetic acid). Mobile phase B (99:1 isopropanol:water + 5 mM ammonium acetate + 0.1% acetic acid). The time program for the pump consisted of a linear gradient as follows: 0 min (100% A; 0% B), 3 min (100% A; 0% B), 5 min (80% A, 20% B), 25 min (70% A, 30% B), 35 min (5% A, 95% B), 36 min (5%A, 95% B), and 38 min (100% A, 0% B). The column was regenerated at initial mobile phase conditions for 3 min after every run.

### Cell viability assays

Cell viability was assessed after the indicated treatment using CellTiter-Blue Reagent (Promega, G8080) or XTT (Sigma-Aldrich, X4626) according to manufacturer’s instructions.

### qPCR

RNA was extracted from cells using TRIzol reagent (Invitrogen, 15596026) according to standard manufacturer’s protocol. Two micrograms of RNA was used as a template for first strand cDNA synthesis using SuperScript II Reverse Transcriptase (Invitrogen, 18064022). qPCR was performed using iTaq Universal SYBR Green Supermix (Bio-Rad, 1725124) and relative mRNA abundance was quantified using a standard curve consisting of pooled cDNA from all samples. TBP was used as a loading control to correct for inaccuracies in pipetting or RNA quantitation.

### TCGA data analysis

ccRCC mRNA expression data and associated clinical outcome data was downloaded from the cBioPortal (www.cbioportal.org) TCGA KIRC data [38]. To determine differential expression levels in ccRCC tumors compared to normal adjacent kidney tissue, mRNA levels were extracted from the corresponding tumor biopsies and normal tissue samples. For survival analyses, patients were segregated based on the median expression of ALOX5 mRNA and day to death or day to last follow up parameters were used to determine Kaplan-Meier overall survival curves.

### Flow cytometry assays

BODIPY 493/503 (Cat#D3922) was purchased from ThermoFisher. BODIPY was diluted at 1 mg/mL in DMSO to make a storage solution. Storage solution was diluted 1:1000 in PBS to make a staining solution. Cells were washed once with PBS pre-staining and then stained by being incubated with staining solution for 15 min at 37 °C. After staining, cells were washed 3 times with PBS before being collected for FACS analysis. For PO-PRO (ThermoFisher Cat# V35123) staining, dye was diluted 1:500 in PBS to make a staining solution. Cells were washed and collected as per usual and then spun down. Cells were re-suspended in staining solution and incubated on ice for 30 min before being spun down and resuspended for FACS analysis. For cleaved caspase 3 FACS assay, anti-cleaved caspase 3 antibody (cell signaling cat#9661) was used as per manufacturer’s instructions. goat anti-rabbit Alexa Fluor 594 (Thermo Fisher, cat#A-11012) was used for secondary antibody staining. FACS analysis was performed using a BD Biosciences LSRFortessa X-20 as per manufacturer’s instructions. Data was analyzed using FlowJo (BD Biosciences).

### Tumor xenografts

All procedures involving animals and their care were approved by the Institutional Animal Care and Use Committee of Stanford University in accordance with institutional and National Institutes of Health guidelines. To establish orthotropic ccRCC xenograft model, Rag2−/−IL2rg−/− double knockout mice aged from 6 and 12 weeks were injected with a 100-μL of collagen plug containing 1× 106 ccRCC cells under the renal capsule as previously described [39]. Briefly, mice were anesthetized and maintained under a heating lamp. The skin at the incision site was shaved and sterilized with three scrubs of povidone iodine followed 70% ethanol. A short incision was made with a scalpel immediately over the kidney. The kidney was gently exposed through the incision and kept moist with sterile physiological saline. A small hole in the renal capsule was made with forceps and raised to create a small pocket between the capsule and the underlying kidney tissue. The 100 μl collagen plug (3.5 mg/mL containing 1 × 106 cells) was inserted into this pocket with forceps. The renal capsule was released and allowed to cover the inserted tissue slice. The kidney was gently placed back through the incision, and the body wall was sutured. The skin incision was sealed with wound clips. Carprofen (4–5 mg/kg) was injected subcutaneously. Sterile practices were followed throughout the surgical procedure. Six weeks after implantation, mice were sacrificed and tumors were dissected and weighted.

### Statistics

Statistics were calculated using Microsoft Excel and GraphPad Prism Software Version 8.0 (GraphPad Prism, San Diego, CA, USA). All statistical tests, n values, statistical significance and any other relevant statistical parameter are indicated in the figure legends. For statistical comparisons, we performed unpaired two-tailed Student’s *t* test, one-way or two-way ANOVAs with either Tukey’s or Šídák’s post-hoc analysis for multiple comparisons where appropriate. A value of *p* < 0.05 was considered significant (represented as *p < 0.05, **p < 0.01, ***p < 0.001, ****p < 0.0001 or not significant (n.s.)).

## Supplementary Information

**Additional file 1: Supplemental Figure 1.** A) Comparison of cholesterol ester levels in RCC4 cells with and without VHL as measured by LCMS. Prior to lipid extraction cells were cultured overnight in media supplemented with CSFBS +/- 100 μM oleic acid. B) FACS analysis of BODIPY 493/503 fluorescent intensity in RCC4 cells transfected with siACSL3 or non-targeting control and cultured in media supplemented with FBS or FBS and 100uM Oleic Acid. C) qPCR results showing mRNA expression levels of ACSL family members in ccRCC tumors compared to normal renal cortex. Data was generated from TCGA KIRC dataset accessed using cBioPortal. Note that ACSL6 was excluded from presentation due to low expression levels in the renal cortex and associated tumor tissue.

**Additional file 2: Supplemental Figure 2.** A) qPCR results showing ACSL3 mRNA levels in parental RCC4 cells and RCC4 cells expressing siRNA resistant ACSL3 and transfected with ACSL3 siRNA or non-targeting control. ACSL3 expression levels were normalized to TBP levels as a loading control. *n*=3, error bars depict standard deviation from the mean. B) Oil Red O images of parental RCC4 cells and RCC4 cells expressing siRNA resistant ACSL3 exposed to 50 uM oleic acid for 18 hrs. *n*=2, images presented at the same magnification. C) qPCR results showing ACSL3 mRNA levels in RCC4 cells transfected with the individual siRNA oligonucleotides from the ACSL3 SMARTpool siRNA that is used throughout the manuscript. *n*=2, error bars depict the standard deviation from the mean. D) qPCR results showing ACSL3 mRNA expression levels in ccRCC cells transfected with shRNA against ACSL3 or scrambled negative control shRNA. *n*=3, error bars depict the standard deviation from the mean. E) Images of Oil Red O stained RCC4 cells transfected with the individual ACSL3 siRNA oligonucleotides from the ACSL3 SMARTpool siRNA. *n* =2, images presented at the same magnification.

## Data Availability

ccRCC mRNA expression data and associated clinical outcome data was downloaded from the cBioPortal (www.cbioportal.org) TCGA KIRC data [38]. The other data used to produce this study is available either within this article or the supplementary figures. Any other data is available from the corresponding author upon reasonable request.

## Abbreviations

ccRCC: Clear cell renal cell carcinoma
5-LOX: 5-lipoxygenase
ACSL: acyl-CoA synthetase
CSFBS: Charcoal stripped fetal bovine serum
HIF: Hypoxia inducible factor
GPX4: Glutathione peroxidase 4
VHL: Von Hippel-Lindau
PUFA: Polyunsaturated fatty acid
DGAT: Diglyceride acyltransferase
