# Supplementary figures and images for: ACSL3 regulates lipid droplet biogenesis and ferroptosis sensitivity in clear cell renal cell carcinoma
Source: Cancer Metab. 2022 Oct 3;10:14. doi: 10.1186/s40170-022-00290-z (PMC9528056; doi:10.1186/s40170-022-00290-z)

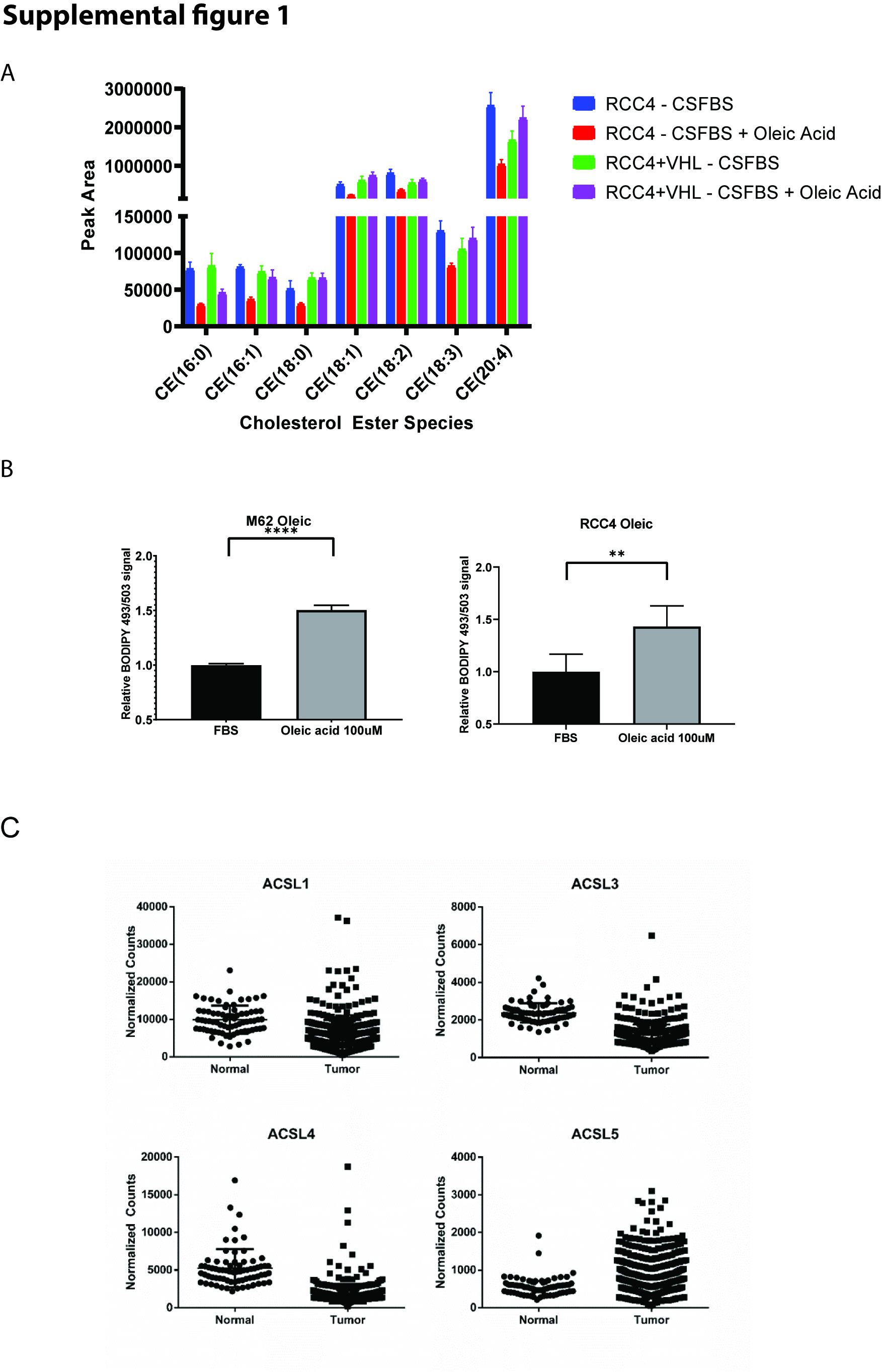

Supplement: Supplementary file 1 — Additional file 1: Supplemental Figure 1. A) Comparison of cholesterol ester levels in RCC4 cells with and without VHL as measured by LCMS. Prior to lipid extraction cells were cultured overnight in media supplemented with CSFBS +/- 100 μM oleic acid. B) FACS analysis of BODIPY 493/503 fluorescent intensity in RCC4 cells transfected with siACSL3 or non-targeting control and cultured in media supplemented with FBS or FBS and 100uM Oleic Acid. C) qPCR results showing mRNA expression levels of ACSL family members in ccRCC tumors compared to normal renal cortex. Data was generated from TCGA KIRC dataset accessed using cBioPortal. Note that ACSL6 was excluded from presentation due to low expression levels in the renal cortex and associated tumor tissue. [file 40170_2022_290_MOESM1_ESM.tif]

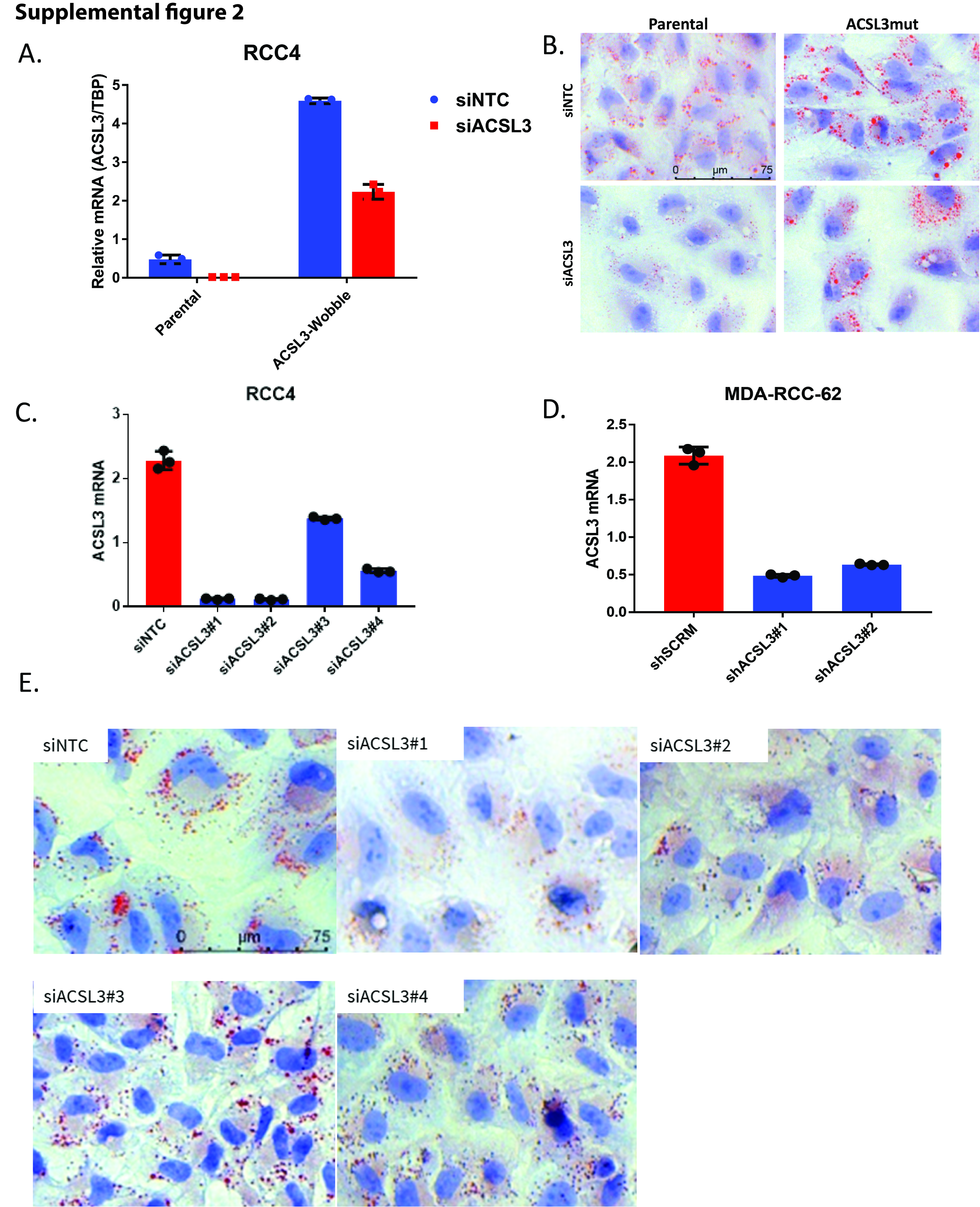

Supplement: Supplementary file 2 — Additional file 2: Supplemental Figure 2. A) qPCR results showing ACSL3 mRNA levels in parental RCC4 cells and RCC4 cells expressing siRNA resistant ACSL3 and transfected with ACSL3 siRNA or non-targeting control. ACSL3 expression levels were normalized to TBP levels as a loading control. n=3, error bars depict standard deviation from the mean. B) Oil Red O images of parental RCC4 cells and RCC4 cells expressing siRNA resistant ACSL3 exposed to 50 uM oleic acid for 18 hrs. n=2, images presented at the same magnification. C) qPCR results showing ACSL3 mRNA levels in RCC4 cells transfected with the individual siRNA oligonucleotides from the ACSL3 SMARTpool siRNA that is used throughout the manuscript. n=2, error bars depict the standard deviation from the mean. D) qPCR results showing ACSL3 mRNA expression levels in ccRCC cells transfected with shRNA against ACSL3 or scrambled negative control shRNA. n=3, error bars depict the standard deviation from the mean. E) Images of Oil Red O stained RCC4 cells transfected with the individual ACSL3 siRNA oligonucleotides from the ACSL3 SMARTpool siRNA. n =2, images presented at the same magnification. [file 40170_2022_290_MOESM2_ESM.tif]
